# Sex identification in Australian skinks (*Egernia stokesii*, *E. hosmeri*, *E. striolata*) using cloacoscopy

**DOI:** 10.17221/3/2023-VETMED

**Published:** 2023-06-20

**Authors:** Zdenek Knotek, Matteo Oliveri, Eva Cermakova, Petr Sramek

**Affiliations:** ^1^Avian and Exotic Animal Clinic, Faculty of Veterinary Medicine, University of Veterinary Sciences Brno, Brno, Czech Republic; ^2^Faculty of Veterinary Medicine, Teaching Veterinary Hospital, University of Teramo, Teramo, Italy; ^3^The Brno Zoological Garden, Brno, Czech Republic

**Keywords:** lizard cloaca, monomorphic reptiles, urodeum, vaginal pouch

## Abstract

Thirteen adult healthy captive skinks, six Gidgee spiny-tailed skinks (*Egernia stokesii*), three Hosmer’s spiny-tailed skinks (*Egernia hosmeri*) and four tree crevice-skinks (*Egernia striolata*), were submitted to the study. The weight of the animals ranged from 28 g to 146 g. All skinks were explored during their putative mating season, which is December. Lizards were restrained manually and positioned in ventral recumbence. Cloacoscopy was performed with the rigid endoscope, protecting channel, endocamera and recording system Telepack-Pal (Karl Storz Endoskope, Tuttlingen, Germany). While the endoscope was gently introduced into the cloaca and advanced into the coprodeum sterile saline was flushed into the cloaca through the protecting channel. The endoscope was then slowly withdrawn to allow visualization of the main structures of the urodeum and proctodeum. Male skinks are characterized by the presence of urethral *papillae* and only one horizontal septum which divides the chamber of the urodeum into two subchambers. Female skinks are characterised by the presence of two septa. The central-dorsal fold that divides the urodeum into two pouches in female skinks is absent in males. Cloacoscopy proved an effective method of sex identification and can be considered a valuable method for breeding and conservation in monomorphic skink lizards.

Sex identification in monomorphic lizard species is challenging. Detailed knowledge of reptile cloacal anatomy is necessary for sex determination and breeding programs ex-situ for endangered reptile species (Fox 1977; Trauth et al. 1987; O’Malley 2005; Oliveri et al. 2016; Oliveri et al. 2018; Spadola et al. 2021; Spadola et al. 2022). Hemipenal pouches probing or hemipenes eversion have been described in snakes and lizards as feasible methods for sexing (Stahl and DeNardo 2019), however, misinterpretations are common in varanids, helodermatids, and skinks. Ultrasound and endoscopy proved to be valuable methods for clinical sex identification in monomorphic lizard species (Schildger and Wicker 1989; Hochleithner and Sharma 2019). Sexual dimorphism of the cloaca has been described in the broad-headed skink, *Eumeces laticeps,* using physical examination and light microscopy, with the most striking differences occurring in the urodeum (Trauth et al. 1987). Males have more prominent urogenital papillae than females, whereas females exhibit well-developed urodeal chambers that are reduced in males. Our previous studies described the cloacoscopy as a feasible method for sex identification in horned vipers and tegu lizards (Oliveri et al. 2016; Morici et al. 2017; Spadola et al. 2022).

Opening in the dorsal urodeum, vaginal pouches have been described in female vipers and tegus (Oliveri et al. 2016). Recently, cloacal anatomy and sex identification with the use of cloacoscopy have been published for another monomorphic lizard, *Tiliqua* sp. (Spadola et al. 2021).

Lizards of the genus *Egernia* are diurnal, viviparous species, endemic to Australia. They are monogamous and form stable, long-term social aggregations. They are omnivorous, eating insects, leaves, plants, and berries. The primary diet of *E. striolata* consists of hard-bodied insects. Potential predators include cats, foxes, dingos, snakes, and birds of prey. Members of genus *Egernia* have moderately-sized heads. Tails are thick and tapering and the tongue is unpigmented. *Egernia stokesii* ranges in colour from olive to reddish brown, with white to yellow ventral scales. *Egernia hosmeri* is reddish-brown on top, with both scattered darker and paler spots along the back, legs, and tail. It has a brown head and neck, a white abdomen, and a few dark brown blotches under the chin. *Egernia striolata* is colored dark-black to grey-brown with a pale stripe going from the head to the tail. The snout-to-vent length (SVL) of a mature *E. stokessi* and *E. hosmeri* is 155–190 mm and 180 mm, respectively. The SVL of a mature *E. striolata* is 100–118 mm. *E. striolata* is highly territorial and tends to defaecate in or by the home site, creating scat piles to mark the territory.

The aim of this study was to validate a cloacoscopic sex identification technique (CSI) in *Egernia stokesii*, *E. hosmeri* and *E. striolata*.

## MATERIAL AND METHODS

**Figure 1:**
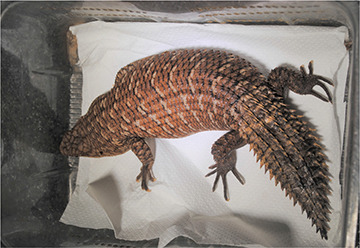
Female Gidgee spiny-tailed skink (*Egernia stokesii*)

**Figure 2:**
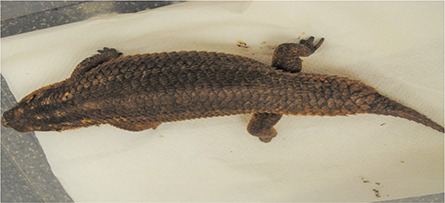
Male Hosmer’s spiny-tailed skink (*Egernia hosmeri*)

Thirteen adult captive skinks of the *Egernia* species were examined: six one-to-three-year-old *E. stokesii* (Figure 1), three eight-year-old *E. hosmeri* (Figure 2), and four one-to-two-year-old *E. striolata* (Table 1).

**Table 1:** Skinks used for cloacoscopy sex identification

Skinks	Species	Body weight (g)	Sex (M/F)*	Age (years)
1	*Egernia stokesii*	146	F	3
2	*Egernia hosmeri*	133	F	8
3	*Egernia stokesii*	126	M	3
4	*Egernia stokesii*	116	M	3
5	*Egernia hosmeri*	128	M	8
6	*Egernia hosmeri*	125	M	8
7	*Egernia stokesii*	70	F	2
8	*Egernia stokesii*	72	M	2
9	*Egernia stokesii*	62	M	1
10	*Egernia striolata*	33	M	2
11	*Egernia striolata*	34	M	2
12	*Egernia striolata*	28	M	1
13	*Egernia striolata*	33	F	2

*Identified by cloacoscopy

The cloacoscopy procedure was performed in compliance with directive 2010/63/EU of the European Parliament and of the Council of the European Union and after ethical approval; with high standards of veterinary care followed. Moreover, in addition, informed consent was signed by the breeders of the skinks. The skinks originated from a professional breeding collection. A complete clinical examination was performed prior to the procedure, with all animals were found to be in good health. The weight of the animals ranged from 28 g to 146 g (85.08 ± 45 g). All skinks were examined during the putative mating season, which is December. A cloacoscopy of the lizards was carried out without sedation or anaesthesia. Animals were gently restrained by using a towel and positioned on an electric heating pad. Cloacoscopy was performed with the rigid endoscope (Hopkins Telescope 2.7 mm diameter, 18 cm length, angle of 30°; Karl Storz Endoskope, Tuttlingen, Germany) and a protecting channel. Images were recorded using an endoscopic camera Telecam (Karl Storz Endoskope, Tuttlingen, Germany) connected to the recording system Telepack-Pal (Karl Storz Endoskope, Tuttlingen, Germany). The endoscope was gently introduced into the cloaca through the vent and advanced 1 cm to 2 cm into the coprodeum. Simultaneously, sterile saline (0.9% NaCl; B. Braun, Melsungen, Germany) was flushed through the protecting channel to clean the optic and dilate the cloacal mucosa. The endoscope was then slowly withdrawn to allow visualization of the main structures of the urodeum and proctodeum.

## RESULTS

**Figure 3:**
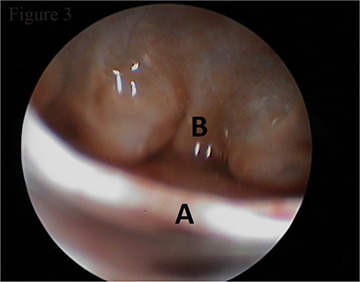
Female tree crevice-skink (*E. striolata*) urodeum All structures of the urodeum are divided by two septa: One separating the sphincter of the coprodeum from the vaginal pouches (A), and the other (B) separating the two vaginal pouches

Cloacoscopy offers excellent imaging of the coprodeum. Retraction of the endoscope in a caudal direction clearly shows the two septa in a female skink, or in the case of a male, one septum. The sphincter of the coprodeum (SC) divides the urodeum from the coprodeum, and it is visible ventrally. Dorsally, two vaginal pouches (VPs) are visible in the urodeum. Compared to other squamata, the VPs are small structures and it is rather difficult to identify them. The urodeum is divided by two septa, one separating the sphincter of the coprodeum from the vaginal pouches (A), and the other (B) separating the two vaginal pouches (Figure 3).

**Figure 4:**
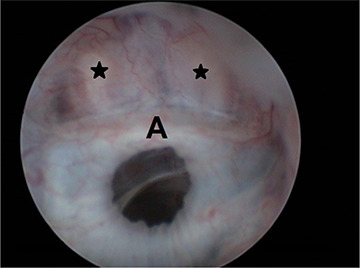
Male tree crevice-skink (*E. striolata*) urodeum Dorsally, the sphincter of the coprodeum is visible (A) The urethral *papillae* (black asterisks) are visible on the dorsal urodeum

The urodeum of female skinks has a central-dorsal fold dividing it into two pouches. In males this is absent. The male urodeum is characterised by the presence of one horizontal septum. Ventrally, the SC is visible. Dorsally, two urethral *papillae* (UP) are visible (Figure 4). Finally, the optic is gently withdrawn from the vent.

## DISCUSSION

A number of methods of sex identification in monomorphic lizard species have been investigated (Morris and Alberts 1996; Di Ianni et al. 2015; Stahl and DeNardo 2019), but many have limitations due to a variety of factors (Philips et al. 2016). Some methods are species-specific, such as the use of radiography for identifying mineralized hemibacula in monitor lizards (*Varanus* spp*.*; Shea and Reddacliff 1986; Phillips et al. 2016) or ultrasonography for identifying the presence of ovarian follicles in adult female viviparous lizards (Gartrell et al. 2002). In the eastern blue-tongued lizard careful manual eversion of the hemipenes would identify a male, but a negative result cannot exclude a male (Phillips et al. 2016). The study by Phillips et al. (2016) is a non-invasive morphometric technique for sex identification in a population of eastern blue-tongued lizards. However, the degree of differences was subtle, requiring the use of ratios. Di Ianni et al. (2015) used ultrasonography, computed tomography, and radiography as methods for sexing eastern blue-tongued lizards. The presence of osteoderms and skin appendages, however, impedes the visualization of the gonads and hemipenes with ultrasonography in *Egernia* spp*.* (Oliveri, personal experience). Other proposed imaging methods such as computed tomography (Morris and Alberts 1996) require sedation and entail prohibitive costs for owners and research institutions. Classical endoscopic methods (coelioscopy) or surgical visualization of gonads are invasive and require anaesthesia and analgesia. Cloacoscopy is a minimally invasive and accurate method of sex identification due to the distinct morphological differences between sexes. Trauth et al. (1987) described in detail the subdivisions within the cloacal complex of the broad-headed skink and the differences between the sexes – the most striking structural modifications occurring in the urodeum. Males had more prominent urogenital papillae than did females, whereas females exhibited well-developed urodeal chambers which were reduced in males.

The existence of similar structures has been described in tegu lizards (Morici et al. 2017; Spadola et al. 2022), male *Tiliqua* sp. (Spadola et al. 2021) and has been recently confirmed also in another monomorphic genus, the helodermatid lizards (our unpublished results).

Female skinks of the Egernia genus (*Egernia hosmeri, E. stokesi, E. striolata*) are characterised by the presence of a central-dorsal fold dividing urodeum in two pouches, which is not found in males. Male skinks are characterised by the presence of one septum which divides the chamber of the urodeum into two sub-chambers. Urethral *papillae* are visible in the dorsal subchamber. These structures and the division of the cloaca into two sub-chambers are major characteristics of the male urodeum in the skinks’ genus *Egernia*.

In conclusion, it could be stated that this study demonstrates that cloacoscopy is a useful technique for sex identification in a monomorphic skink genus, *Egernia*, even in small species (*E. striolata*). Furthermore, cloacoscopy can be considered to be used in the breeding and conservation of endangered skink species.
